# Optimizing YouTube for Medical Education: An Analysis of Popular Cardiovascular United States Medical Licensing Examination (USMLE) Step 1 Videos

**DOI:** 10.7759/cureus.107826

**Published:** 2026-04-27

**Authors:** Varun Hariharan, David Harbaugh, Jay Desai, Benjamin C Beauchamp, Raghuram V Reddy, Thomas K King, Marcia Varella

**Affiliations:** 1 College of Medicine, Florida International University, Herbert Wertheim College of Medicine, Miami, USA; 2 Department of Medical and Population Health Sciences Research, Florida International University, Herbert Wertheim College of Medicine, Miami, USA

**Keywords:** cardiovascular system, content delivery style, e-learning, medical education, usmle step 1, youtube

## Abstract

Background

Electronic learning (e-learning) is an important component of medical education, improving learner engagement and satisfaction. YouTube (Google LLC, Mountain View, CA) is a widely used educational platform due to its accessibility, low cost, and variety of content delivery styles. However, viewer preferences for different content delivery styles across medical education topics remain poorly characterized. Identifying popular delivery styles by topic may inform how educators curate and recommend supplemental videos.

Objective

This study's objective is to describe the content delivery styles and engagement metrics of the most popular YouTube videos across five United States Medical Licensing Examination (USMLE) Step 1 cardiovascular content areas (anatomy, embryology, physiology, pathology, and pharmacology) and to assess whether the distribution of delivery styles varied by topic. Engagement was measured using publicly available popularity indicators (views, likes, comments) rather than direct measures of educational effectiveness.

Methods

In this cross-sectional, observational study, we searched YouTube via Google Chrome (Google LLC, Mountain View, CA) in incognito mode on November 5, 2024. For each topic, results were filtered by "Video" type and sorted by "Most Viewed," and the top 30 videos per content area were included (N = 150). Two pairs of authors independently collected engagement metrics, channel characteristics, and delivery-style classifications; a third author adjudicated discrepancies. Descriptive statistics summarized delivery-style distributions; a Pearson chi-square test assessed the association between topic and delivery style, and Kruskal-Wallis tests compared engagement across styles within each topic.

Results

We assessed 150 videos. Whiteboard-style videos were the most common format in embryology (14, 46.7%), physiology (14, 46.7%), pathology (11, 36.7%), and pharmacology (18, 60.0%), while in anatomy, whiteboard (nine, 30.0%) and animation (eight, 26.7%) styles were used in similar proportions. No statistically significant association was observed between topic and delivery style (Pearson χ²(24) = 27.72, p = 0.272), and Kruskal-Wallis tests did not detect statistically significant differences in median likes across delivery styles within any topic (all p > 0.05). Descriptive patterns suggested that animation had the highest mean views in anatomy, physiology, and pathology, and that lecture-style videos had the highest mean views in pharmacology, but these patterns were not statistically significant and should be interpreted cautiously.

Conclusions

Among the most-viewed YouTube videos in USMLE Step 1 cardiovascular topics, no single delivery style was universally preferred, and engagement patterns varied descriptively by content area. Because our analysis relied on publicly available popularity indicators rather than validated measures of learning, these findings should be interpreted as hypothesis-generating rather than as evidence of educational effectiveness. The results may inform future studies that directly compare delivery styles using standardized content and knowledge-outcome measures.

## Introduction

Medical education has been traditionally conducted through textbooks and in-person lectures. Yet, in recent years, education has changed progressively with the growing use of internet technologies to enhance knowledge and performance, a phenomenon known as electronic learning (e-learning). Nearly two decades ago, researchers recognized the potential of e-learning to enhance medical training [1].

The benefits of e-learning as a complement to traditional learning became increasingly apparent, as it allowed learners to control their learning pace and customize their experience. It has since been shown that online learning experiences result in improved learning outcomes and high student satisfaction [2]. The growth of the internet has greatly increased the number of online resources available to medical students. Specifically, a global video-sharing platform, YouTube (Google LLC, Mountain View, CA), has been shown to be effective in teaching a multitude of aspects of medical education, from basic anatomy to sophisticated surgical procedures [3,4].

Today, YouTube is the largest internet video-sharing website and one of the leading platforms for free medical education content, including convenient feedback features such as comments and likes [5]. Being able to interact directly with content creators allows for all of the advantages of in-person teaching styles while maintaining the benefits of asynchronous learning. These features also provide a quantitative metric to assess the quality and effectiveness of videos.

Although there is evidence that YouTube has benefits for medical education, more research needs to be done to understand the needs of students and how to optimize its use [6,7]. Current research regarding YouTube as a source for medical content aims at assessing user satisfaction, but scarce research exists on the actual content and characteristics of the videos. Evaluating characteristics of the learning material, such as video duration and content-delivery style, may affect engagement metrics and can lead to improved understanding and optimization of those resources for learners. Therefore, this cross-sectional study describes the characteristics of the most popular YouTube videos aimed at preparing medical students for the United States Medical Licensing Examination (USMLE) Step 1 cardiovascular content area and examines whether the distribution of content-delivery styles varies by topic. Importantly, we focus on publicly available engagement indicators (views, likes, comments) as proxies for popularity, rather than on direct measures of learning outcomes, and our findings should be interpreted within this scope.

## Materials and methods

Design

This was a cross-sectional, observational study of publicly available YouTube videos providing medical-education content related to cardiovascular topics relevant to the USMLE Step 1 examination. No human subjects were involved; the analysis relied solely on publicly available platform metadata.

Sample

We selected publicly accessible YouTube videos addressing five cardiovascular medical-education topics aligned with the USMLE Step 1 content outline: cardiovascular embryology, cardiovascular anatomy, cardiovascular physiology, cardiovascular pathology, and cardiovascular pharmacology. Specific search terms were derived from First Aid for the USMLE Step 1 and entered into the YouTube search bar as “Cardiovascular (Content Area).” Results were filtered by "Video" type and sorted by "Most Viewed" to prioritize general popularity metrics. All searches were performed using Google Chrome (Google LLC, Mountain View, CA) in incognito mode to minimize personalized search bias. The top 30 videos for each topic (total N = 150) were included. Videos were excluded if they were promoted through YouTube’s advertising algorithms, were YouTube Shorts under 60 seconds (due to insufficient educational depth), or contained non-educational content focused on entertainment or personal experiences rather than instructional material. 

Because YouTube rankings are shaped by a dynamic and partially personalized recommendation and search algorithm, identical future searches may not retrieve identical result sets. To reduce this variability, all searches were conducted on a single date (November 5, 2024) in incognito mode; nonetheless, the inherent algorithmic dynamics of the platform place an upper bound on the reproducibility of our results, which we acknowledge as a methodological limitation. 

Variables and data collection

Two pairs of authors collected data independently on the same day and time, entering each data point in duplicate to ensure accuracy and consistency. A third author resolved inconsistencies in the data entries. Screenshots of each video title, view count, likes, and comments were taken. The data were entered into a standardized Microsoft Excel (Microsoft® Corp., Redmond, WA) document for real-time updates and cross-validation among team members. Although delivery-style classifications were reviewed by multiple investigators and resolved by consensus, a formal inter-rater reliability metric such as Cohen’s kappa was not calculated. Therefore, some degree of subjective interpretation in content classification may have been present. 

Data collection included explanatory factors and outcome measures. Explanatory factors included video topic (embryology, anatomy, physiology, pathology, or pharmacology), video duration (categorized as under four minutes, four to 20 minutes, or over 20 minutes), date of publication, channel initiation date, number of channel subscribers, whether the video was part of a series, and primary language. Content-delivery style was categorized by the research team through visual review of each video. Styles were defined as lecture-style, whiteboard-based, animated, case-based learning, simulated patient interaction, podcast-style, practical demonstration, live stream, or mixed-style (integrating multiple teaching styles, with each secondary style comprising ≥20% of total video length). Outcome measures assessed video popularity and engagement. We defined popularity primarily by the total number of views, and engagement by the number of likes, comments, and creator replies to the top 10 comments. Two derived metrics, the like-to-view ratio and comment-to-view ratio, were calculated as proxies for audience interaction intensity. These indicators reflect viewer behavior on the platform and should not be interpreted as direct measures of learning or educational effectiveness. 

**Figure 1:**
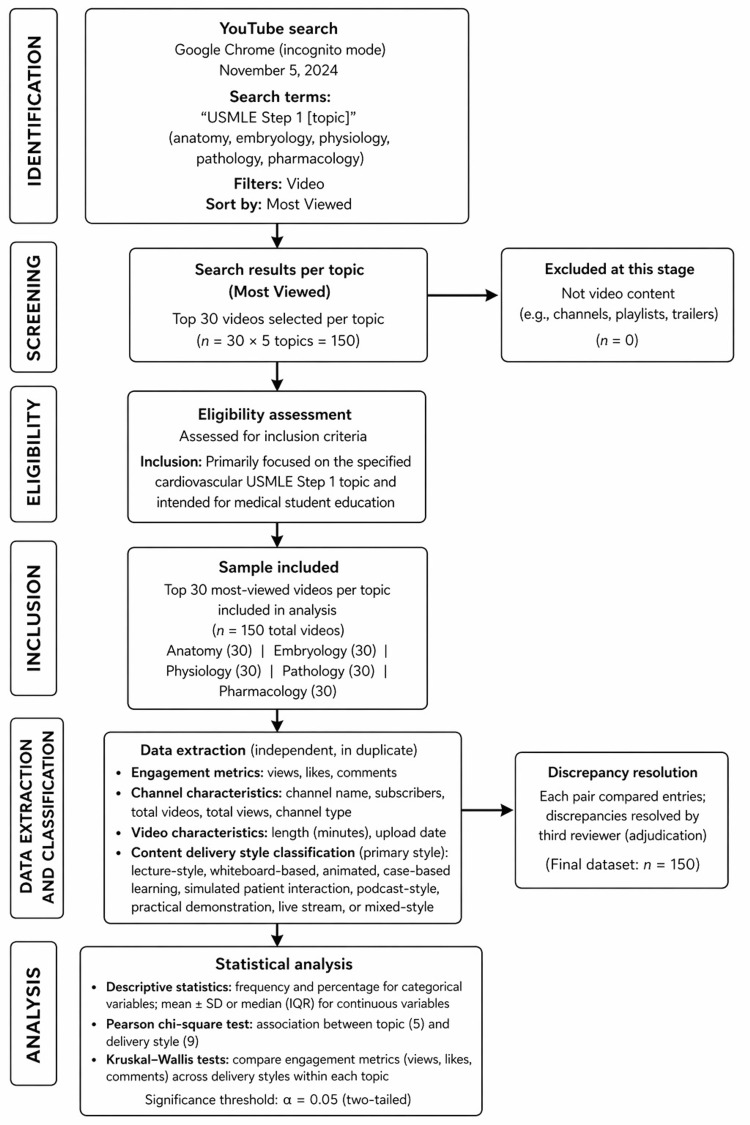
Study workflow for identification, screening, inclusion, data extraction, and analysis of the top 30 most-viewed United States Medical Licensing Examination (USMLE) Step 1 YouTube videos across five content areas

A study flow summarizing the search, screening, and classification process is provided in Figure 1. 

Analysis plan

Descriptive statistics (mean, median, range, and percentiles) were calculated for all engagement metrics. A Pearson chi-square test was used to assess the association between the cardiovascular topic and content delivery style. Because engagement variables (views, likes, comments) were non-normally distributed, comparisons across delivery styles within each topic were performed using the Kruskal-Wallis rank-sum test. Given the exploratory nature of the analysis and the modest within-topic cell counts for several delivery styles, results are interpreted descriptively, and non-significant patterns are not emphasized as comparative findings. All analyses were conducted using Stata version 17 (StataCorp LLC, College Station, TX) [8].

## Results

Descriptive characteristics

**Figure 2:**
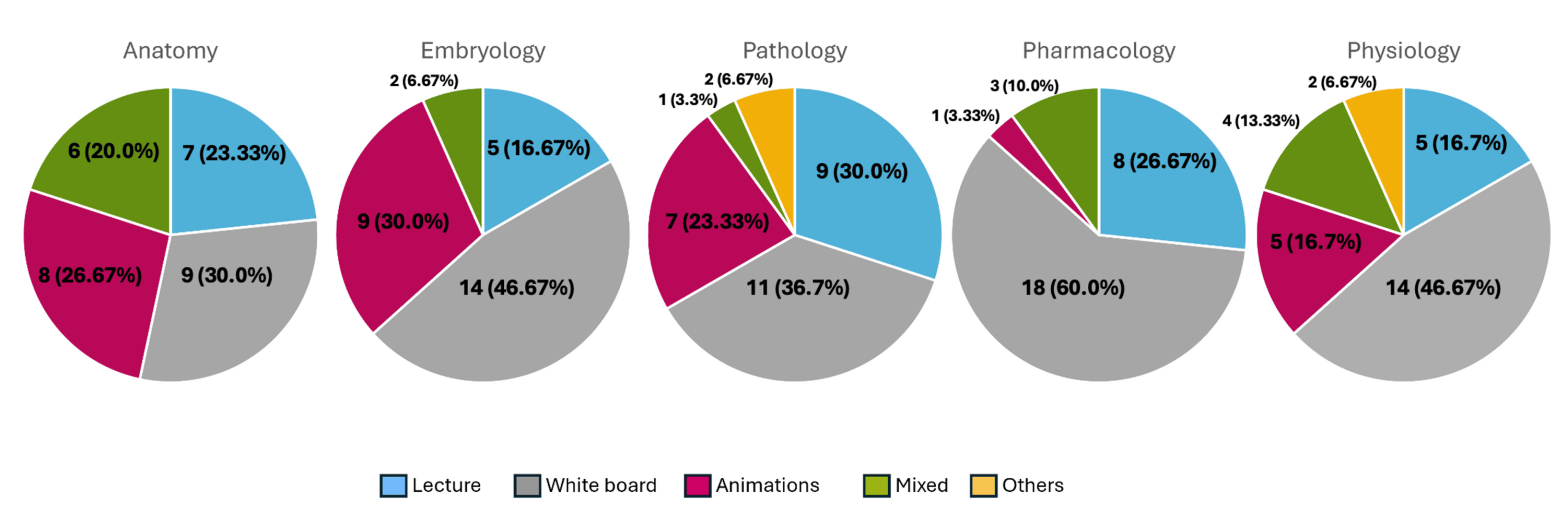
Distribution of delivery methods in the 30 most popular videos for each of the five cardiovascular-system-related content areas

A total of 150 videos, 30 for each of the five medical-education topics (anatomy, embryology, pathology, pharmacology, and physiology), were analyzed. The most common content-delivery styles were whiteboard (66, 44%) and lecture (34, 22.7%), followed by animation (30, 20%), mixed approaches (16, 10.7%), and other styles such as live/interactive sessions, podcast-style discussions, and practical demonstrations (four, combined 2.7%) (Table 1). Whiteboard videos were particularly prevalent in pharmacology (18, 60%), physiology (14, 46.7%), and embryology (14, 46.7%), while lecture styles were commonly used in pharmacology (eight, 26.7%), pathology (nine, 30%), and anatomy (seven, 23.3%) (Figure 2). Physiology had the most diverse range of delivery methods, including live and practical styles. The Pearson chi-square test did not detect a statistically significant association between topic category and content-delivery style (χ²(24) = 27.72, p = 0.272).

**Table 1:** Primary descriptive characteristics of YouTube videos on cardiovascular topics in medical education by content delivery style Abbreviations and symbols: % = percentage; ± = plus or minus; ≥ = greater than or equal to; χ² = chi-square test statistic; df = degrees of freedom; N = total number of videos; p = probability value; SD = standard deviation χ² values represent the Kruskal-Wallis test statistics comparing median likes across content delivery styles within each topic, with corresponding p-values reported.

Category	Content Delivery Style	n (%)	Views Mean ± SD	Views Median	Comments Median	Likes Median	Likes/Views Mean	Comments/Views Mean	Kruskal-Wallis χ² (df)	p-value
Embryology	Whiteboards	14 (46.67%)	528,505 ± 442,613	345,796	376.5	9,000	0.023322	0.000992	4.544 (3)	0.2084
	Animations	9 (30.0%)	452,729 ± 501,009	243,796	119	5,400	0.020493	0.000455		
	Lecture style	5 (16.67%)	445,394 ± 181,850	498,352	152	8,800	0.017842	0.000425		
	Mixed	2 (6.67%)	193,071 ± 49,181	193,071	64	1,815	0.008477	0.000312		
Anatomy	Whiteboards	9 (30.0%)	1,562,871 ± 1,775,922	1,107,725	257	11,000	0.01712	0.000389	7.360 (3)	0.0613
	Animations	8 (26.67%)	4,037,131 ± 2,530,303	4,348,580	1107	49,500	0.016441	0.000468		
	Lecture style	7 (23.33%)	1,102,886 ± 564,985	926,304	397	24,000	0.019611	0.000557		
	Mixed	6 (20.0%)	3,533,626 ± 2,046,282	3,353,334	734	35,000	0.012954	0.000352		
Physiology	Whiteboards	14 (46.67%)	1,603,900 ± 1,782,410	1,071,193	626	22,000	0.021618	0.000699	5.488 (4)	0.2408
	Animations	5 (16.67%)	2,437,024 ± 1,520,611	2,537,839	772	49,000	0.017154	0.000279		
	Lecture style	5 (16.67%)	428,978 ± 297,250	261,258	373	8,300	0.025645	0.000995		
	Mixed	4 (13.33%)	2,214,009 ± 3,088,242	901,817	803	12,000	0.018503	0.000576		
	Live streams and interactive sessions	1 (3.33%)	2,162,513	2,162,513	429	27,000	0.012485	0.000198		
	Practical skills demonstrations	1 (3.33%)	177,425	177,425	106	1,100	0.0062	0.000597		
Pathology	Whiteboards	11 (36.67%)	923,607 ± 665,171	641,882	298	9,600	0.01583	0.000499	3.749 (4)	0.4411
	Animations	7 (23.33%)	1,199,345 ± 1,160,143	754,074	264.5	6,800	0.010868	0.000325		
	Lecture style	9 (30.0%)	687,694 ± 527,470	395,009	127	5,750	0.01342	0.000362		
	Mixed	1 (3.33%)	674,674	674,674	164	12,000	0.017786	0.000243		
	Podcast-style videos	1 (3.33%)	448,212	448,212	580	6,400	0.014279	0.001294		
	Live streams and interactive sessions	1 (3.33%)	332,268	332,268	212	5,800	0.017456	0.000638		
Pharmacology	Whiteboards	18 (60.0%)	710,167 ± 431,448	503,916	428	9,350	0.018698	0.000851	6.131 (3)	0.1054
	Animations	1 (3.33%)	321,127	321,127	62	5,800	0.018061	0.000193		
	Lecture style	8 (26.67%)	1,016,647 ± 577,323	897,459	228	13,500	0.017475	0.000345		
	Mixed	3 (10.0%)	467,058 ± 185,707	409,713	205	6,500	0.017215	0.000466		
Total	Whiteboards	66 (44.00%)	1,013,064 ± 1,172,127	641,393	389	10,000	0.019604901	0.00072692	-	
	Animations	30 (20.00%)	1,909,109 ± 2,091,926	930,752	291	13,000	0.016528793	0.0003915		
	Lecture style	34 (22.67%)	776,897 ± 533,426	570,846	223	12,000	0.01823843	0.00050454		
	Mixed	16 (10.67%)	2,032,486 ± 2,298,319	674,674	347.5	12,000	0.014640946	0.00041724		
	Podcast-style videos	1 (0.67%)	448,212	448,212	580	6,400	0.01427896	0.00129403		
	Live streams and interactive sessions	2 (1.33%)	1,247,391 ± 1,294,179	1,247,391	320.5	16,400	0.014970631	0.00041821		
	Practical skills demonstrations	1 (0.67%)	177,425	177,425	106	1,100	0.0061998	0.00059744		

Primary outcomes

**Figure 3:**
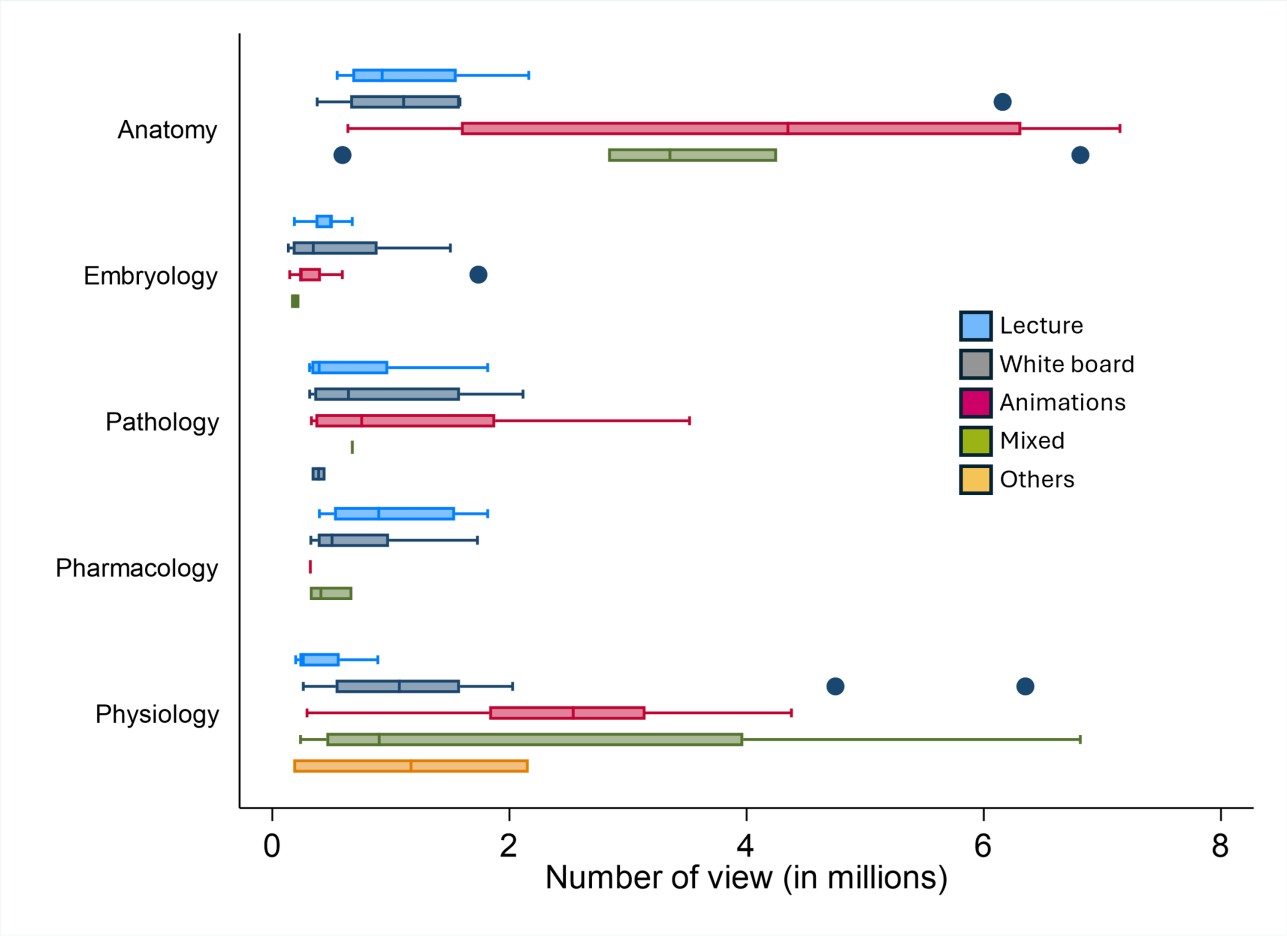
Distribution of the number of views of the top 30 videos according to delivery methods in five cardiovascular-system-related content areas

**Figure 4:**
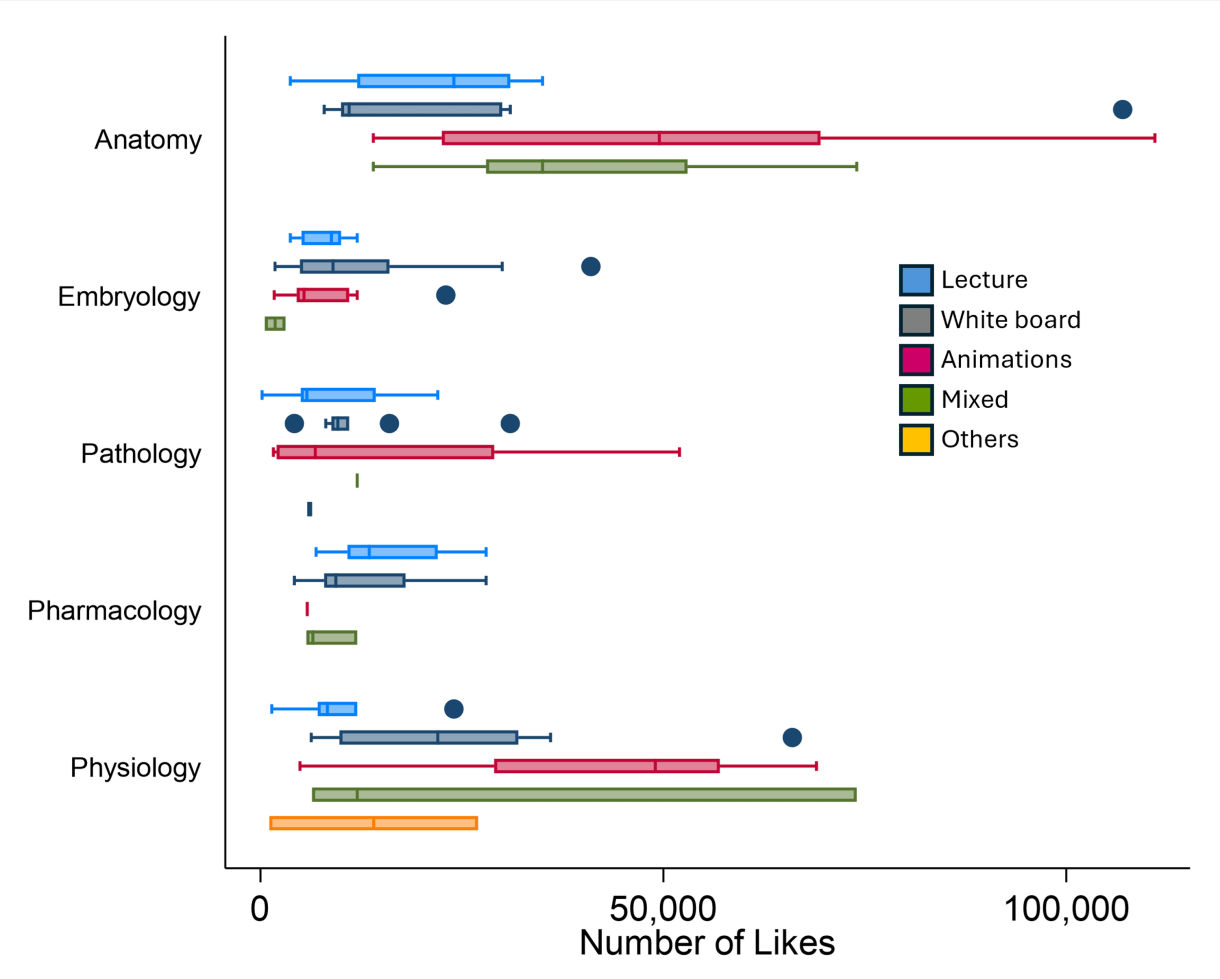
Distribution of the number of likes of the top 30 videos according to delivery methods in five cardiovascular-system-related content areas

The distribution of views and likes according to the teaching method used is shown for each cardiovascular topic (Figure 3 and Figure 4). Kruskal-Wallis tests did not detect statistically significant differences in median likes across delivery styles within any cardiovascular topic (all p > 0.05; Table 1). Given these non-significant results, the following patterns are presented as descriptive observations only and should not be interpreted as comparative evidence that one delivery style outperforms another. 

Descriptive comparisons showed that lecture-style videos had the highest median likes in pharmacology (13,500; p = 0.1054) but comparatively lower median likes in physiology (8,300; p = 0.2408). In anatomy, animation was associated with the highest median likes (49,500; p = 0.0613) and high mean view counts (4,037,131 ± 2,530,303) (Figure 4). In pathology, mixed-format videos had the highest median likes (12,000; p = 0.4411), whereas in embryology, whiteboard videos had the highest median likes (9,000; p = 0.2084). While whiteboard videos were the most frequent format overall, accounting for 66 (44%), mean views ranged from 528,505 in embryology to 1,603,900 in physiology. These numeric differences are reported for transparency but did not reach statistical significance. 

Secondary outcomes

Several secondary descriptive patterns were observed. Videos presented in English had a greater median like count. Videos that were part of a series also showed greater engagement, with greater median views and likes compared to standalone videos. Videos in the four- to 20-minute range accounted for the majority of high-view videos in our sample; we present this as a descriptive observation and did not perform a formal inferential test of duration on engagement. Days since upload did not show a significant correlation with total views, suggesting that video age alone was not a reliable predictor of popularity. Taken together, these secondary observations suggest that video language, structural design, and continuity (e.g., series-based learning) may be worth exploring in future work, even in the absence of a universally superior delivery style. 

## Discussion

Interpretation of findings

This cross-sectional study described the delivery styles and engagement metrics of the most popular YouTube videos for USMLE Step 1 cardiovascular topics and examined whether the distribution of delivery styles varied by topic. Overall, no single style was found to be associated with being superior to others in terms of popularity. Rather, the popularity of a video style varied depending on the content area. 

Previous studies have shown that video-based learning, such as YouTube, is effective for educational delivery [9-13]. Multimedia and animation styles have increased learner engagement and improved knowledge retention when compared to lecture-style [14-16]. Much of the existing literature has not evaluated the type of educational delivery styles on YouTube through viewer preferences. Our study provides preliminary new insights by using a structured approach, categorizing delivery styles and secondary characteristics through their relative popularity within unique cardiovascular topics. Traditionally, lecture-style instruction is the standard educational delivery style, suggesting that this style is preferred across all content areas. However, our study found that subject areas within the cardiovascular system were associated with varied descriptive patterns of content-delivery-style use. These findings suggest that descriptive preferences for educational video styles may be content-dependent; however, because the observed differences did not reach statistical significance, they should be viewed as hypothesis-generating rather than as definitive evidence. 

The increased prevalence of whiteboard videos in embryology and pathology and the increased popularity of animation in anatomy could be attributed to dual-coding theory. Dual-coding theory suggests that humans process information through two independent processes that are interconnected: a verbal system and a nonverbal system [17]. Learning is improved when engaging both systems simultaneously, since two retrieval pathways are active for a single topic rather than either pathway alone [17]. For example, embryology involves a spatial and temporal developmental process that can be better understood through verbal explanation paired with simplified sketches. Similarly, anatomy and pathology require learners to understand spatial relationships and how structures interact mechanically, triggering a visual retrieval pathway that is reinforced by verbal instruction. When a pathological event occurs, learners will utilize imagery to envision the change in tissue architecture. On the other hand, physiology and pharmacology require more abstract thinking. Physiology is associated with fixed anatomy but requires more concept-driven cognitive processing to appreciate the dynamic processes, such as feedback loops. Likewise, pharmacology encourages conceptual thinking through receptor pathways and dose-response relationships. Research on dual-coding theory demonstrates that presenting information both verbally and visually enhances retention [18,19]. The dual-coding theory suggests how a dual-sensory type of style may be beneficial for learners within the areas of embryology, pathology, and anatomy. 

Complementing dual-coding theory, the learning pyramid framework (often attributed to Edgar Dale’s Cone of Experience and popularized in later iterations) posits that more passive modalities, such as lectures, generally yield lower retention than more active or self-directed learning modes [20]. Although the specific retention percentages commonly cited with the learning pyramid have been critiqued for lacking rigorous empirical support [20], the broader concept - that self-directed, multimodal engagement tends to promote deeper learning than passive listening - is consistent with our finding that learners on YouTube gravitate toward formats (e.g., whiteboard, animation) that pair narration with active visual construction rather than toward pure recorded lectures. In this framing, YouTube functions as a self-directed supplement to traditional lecture-based curricula, and the predominance of visually active formats in our sample aligns with the pyramid’s emphasis on active, learner-controlled engagement. 

The rapid development of artificial intelligence (AI) in medical education also offers potential avenues to enhance the creation and curation of e-learning videos, such as those studied here. Large language models and generative-AI tools have been evaluated for content generation, personalization, and formative assessment in medical training [21,22], and early work has documented AI performance on standardized medical examinations, including the USMLE [23]. In the context of short-form educational video, AI could assist creators with drafting scripts, producing animated visualizations of anatomic or physiologic processes, auto-generating searchable transcripts and chapter markers, and tailoring video length and difficulty to the viewer's level. AI-driven analytics on viewer behavior may also help educators identify which delivery styles resonate with particular learner populations and could inform evidence-based curation of supplemental YouTube content. These applications are promising but require careful validation for factual accuracy, bias, and alignment with curricular standards before routine adoption.

Strengths and limitations 

Strengths of this study include the large number of videos assessed (150 YouTube videos across five content areas), using a standardized and reproducible search methodology, and reducing selection, procedural, and observer biases. The data were independently collected by two pairs of authors and cross-validated by a third to resolve discrepancies, enhancing the study’s replicability and validity.

Several limitations temper the interpretation of our findings and should be acknowledged explicitly. First, our analysis is based entirely on publicly available engagement indicators (view counts, likes, comments), which serve as proxies for popularity and not as direct measures of educational effectiveness; whether the observed delivery styles are associated with better knowledge acquisition or long-term retention remains unknown. Second, the reproducibility of our YouTube searches is inherently limited by the platform’s dynamic, partially personalized ranking algorithm; despite using incognito mode on a single date, identical future searches may not retrieve identical result sets. Third, our sample includes only the top 30 most-viewed videos per topic. This deliberately selects for high-viewership content and introduces a selection bias: we did not compare these popular videos with less-viewed or potentially lower-quality videos, nor did we compare videos of equivalent instructional content that differ only in delivery style, which would be necessary to draw causal conclusions about style per se. Fourth, creator-level factors - for example, a creator’s technical ability to produce animations, their production budget, or their platform following - likely influence both style and popularity and are not captured in our analysis. Fifth, we did not compute a formal inter-rater reliability coefficient (e.g., Cohen’s kappa) for the delivery-style classification; instead, we relied on duplicate independent extraction by two author pairs with third-author adjudication. Sixth, we lacked information on viewer-level characteristics, such as the level of training or native language, because these data are not available through public YouTube metadata. Although previous studies have similarly used engagement metrics to evaluate learner preferences [24-26], the limitations noted above should be considered when interpreting our results.

Implications for medical educators 

Despite these limitations, our findings suggest three cautious implications for medical educators. First, the high engagement observed across videos suggests that learners frequently rely on YouTube as a supplemental resource for cardiovascular topics, highlighting its relevance for educators when considering supplemental materials. Second, because no single delivery style was associated with superior engagement across all topics, we suggest that supplemental YouTube videos should be tailored by delivery style to the content area (e.g., pharmacology, physiology). For example, animation-type videos may be considered for anatomy and lectures for pharmacology. Third, our descriptive observation that videos of four to 20 minutes accounted for most high-view content is consistent with a preference for shorter, more focused supplemental videos and may inform how educators structure video series. 

Future research 

Future research should move beyond popularity indicators and directly assess how video style affects actual learning outcomes, such as immediate, short-term, and long-term retention. Additionally, analyzing the educational level of each video can aid in understanding the engagement. More advanced videos may provide greater topic depth, but can be more challenging for certain audiences, possibly deterring them while attracting those with advanced education. Controlled comparisons of videos covering equivalent content but differing in delivery style, combined with learner-level demographic data, would allow stronger causal inference than is possible in platform-based observational designs such as ours. AI-assisted content generation and analytics, discussed above, represent a further promising direction for both producing and evaluating next-generation supplemental videos.

## Conclusions

In this cross-sectional descriptive study of the 150 most-viewed YouTube videos for USMLE Step 1 cardiovascular topics, no single content-delivery style was universally preferred across subject areas. The distribution of delivery styles did not differ significantly by topic, and within-topic engagement differences between delivery styles did not reach statistical significance. The descriptive patterns we observed - for example, higher mean views for animation-style videos in anatomy and for lecture-style videos in pharmacology - should be regarded as hypothesis-generating rather than as definitive evidence that a specific style is superior.

Because our analysis relied on publicly available popularity indicators rather than validated measures of learning, we caution against interpreting high engagement as evidence of educational effectiveness. Nonetheless, the clear and broad use of YouTube by learners underscores the importance of thoughtful curation of supplemental video content. Educators may consider matching delivery style to content area, favoring shorter, focused videos within a coherent series, and critically evaluating new AI-assisted tools for producing and personalizing supplemental educational content, while future controlled studies examine the link between delivery style and learning outcomes.
